# RNA-seq data of *Elaeidobius kamerunicus* from North Sumatera and Central Kalimantan in Indonesia

**DOI:** 10.1016/j.dib.2023.109816

**Published:** 2023-11-17

**Authors:** Agus Eko Prasetyo, Edhi Martono, Andi Trisyono, Alan Soffan

**Affiliations:** a Department of Plant Protection, Faculty of Agriculture, Universitas Gadjah Mada, Yogyakarta, Indonesia; b Research Centre for Biotechnology, Universitas Gadjah Mada, Yogyakarta, Indonesia; c Department of Crop Protection, Indonesian Oil Palm Institute, Medan, Indonesia

**Keywords:** *Elaeidobius kamerunicus*, Oil palm, Pollinator, RNA-seq, Transcriptomic data

## Abstract

Since introduced from West Africa at 40 years ago, *Elaeidobius kamerunicus* Faust. (Coleoptera: Curculionidae) is still the main pollinator agent of oil palm plantation in Indonesia until now. Unfortunately, the success rate of pollination in various regions in Indonesia is relatively different, for example in Sumatra and Kalimantan. The oil palm fruit set formed in Kalimantan tends to be lower than in Sumatra. Preliminary studies show that weevils from Kalimantan visit female flowers less than from Sumatra. However, the molecular mechanisms involved in regulating insect behavior, especially in their role as pollinating agents, are not yet clearly understood. Therefore, a transcriptomic study was carried out to obtain raw data to determine gene expression differences in studying the behavior of the same weevil from two different regions. Here, we present two data sets of RNA seq reads which are available in GenBank Sequence Read Archive (SRA) database with accession number of SRR21521626 and SRR21521625 for weevil from North Sumatra and Central Kalimantan respectively.

Specifications Table

**Table:** 

Subject	Insect science
Specific subject area	Trancriptomics
Type of data	Transcriptome sequences (RNA-Seq raw reads)
How data were acquired	Illumina Novaseq 6000 PE150 sequencing platform
Data format	Raw sequences (FASTQ)
Parameter for data collection	*Elaeidobius kamerunicus* from North Sumatera (EkNS) and from Central Kalimantan (EkCK)
Description of data collection	EkNS and EkCK weevil samples (whole body) were collected from North Sumatra and Central Kalimantan respectively. Total RNA was isolated and cDNA libraries were prepared for RNA-sequencing. The RNA-seq raw reads were further analyzed to get the clean reads and stored in FASTQ file.
Data source location	Marihat Estate, PT Perkebunan Nusantara, North Sumatera, Indonesia, GPS data: 2^o^54’22”N, 99^o^6’5”E and Pundu Estate, Bumitama Gunajaya Agro, Central Kalimantan, Indonesia, GPS data: 1^o^59’44”S, 113^o^3’18”E
Data accessibility	Raw data (FASTQ) of EkNS and EkCK has been deposited in NCBI Sequence Read Archive (SRA) data base with the accession numbers SRR21521626 (https://www.ncbi.nlm.nih.gov/sra/SRR21521626) and SRR21521625 (https://www.ncbi.nlm.nih.gov/sra/SRR21521625) respectively

## 1 Value of the Data

• *Elaeidobius kamerunicus* is the main oil palm pollinator in Indonesia [1], [2], [3], however their effectiveness among different region especially in Sumatra and Kalimantan was relatively different [4], [5], [6].
• There are some studies about genomic data of *E. kamerunicus* [7], [8], [9] but not yet for the transcriptomic analysis.
• Two set of raw-FASTQ file transcriptome data, EkNS and EkCK, were reported here to support the understanding of molecular mechanism underlying the behavior of the weevil as oil palm pollinator.
• The transcriptomic data can be further analyzed by examining the differential genes expression which is essential in determining the major genes involved in the behavior of the insect which finally may support the efficiency of oil palm pollination.

## 2 Objectives

Primary objective for this reported data was to support the understanding of molecular mechanism underlying the behavior of *E. kamerunicus* as oil palm pollinator in Indonesia. Second objective was to know the differential genes expression between the weevils from two regions in Indonesia, EkNS and EkCK, especially that involved in behavior as oil palm pollinator insect.

## 3 Data Description

The different oil palm fruit set value in Sumatra and Kalimantan was due to the difference in behavior of *E. kamerunicus* weevil as main oil palm pollinator insect. FASTQ raw data file which was generated from two sets of EkNS and EkCK weevil's transcriptome has been deposited to NCBI-SRA data base with the accession number SRR21521626 and SRR21521625 respectively. These data could be used as guidance for understanding the molecular behavior mechanism of the weevils. Descriptive statistics on the RNA-seq data of the two set of both EkNS and EkCK weevils were given in Table 1.

**Table 1:** Descriptive information for RNA seq raw data for two samples of *Elaeidobius kamerunicus* from North Sumatra (EkNS) and from Central Kalimantan (EkCK).

Descriptive	Sample	
	EkNS	EkCK
Total Raw Reads (Mb)	65.33	43.92
Total Clean Reads (Mb)	63.58	43.73
Total Clean Bases (Gb)	9.54	6.56
Clean Reads Q20 (%)	96.88	97.29
Clean Reads Q30 (%)	91.93	92.40
Clean Reads Ratio (%)	97.33	99.58
Biosample ID	SAMN30696137	SAMN30696138

Total Raw Reads (Mb): The reads amount before filtering.

Total Clean Reads (Mb): The reads amount after filtering.

Total Clean Bases (Gb): The total base amount after filtering.

Clean Reads Q20 (%): The rate of bases which quality is greater than 20 value in clean reads.

Clean Reads Q30 (%): The rate of bases which quality is greater than 30 value in clean reads.

Clean Reads Ratio (%): The ratio of the amount of clean reads.

## 4 Experimental Design, Materials and Methods

### 4.1 Insect sample collection

The collected samples were weevils that had just emerged from the male inflorescence 4-5 days through anthesis [10]. Oil palm male inflorescence was the only one of breeding site for this insect from eggs, larvae to pupae [11,12]. There were two origin places of the weevils i.e., from Marihat, Simalungun, North Sumatra and Pundu, East Kotawaringin, Central Kalimantan. From each place, 3 male inflorescences were taken from 3 locations in one estate, then 10 male and 20 female of new emerged weevils were selected from each inflorescence. Samples for each region were a mixture of both male and female weevils from the three sampling locations. The live weevil's samples were immersed on RNA later (Sigma) into a 1.5 mL tube and then stored at -20 °C for RNA extraction.

### 3.2 RNA isolation, library preparation and RNA-seq

**Fig. 1:**
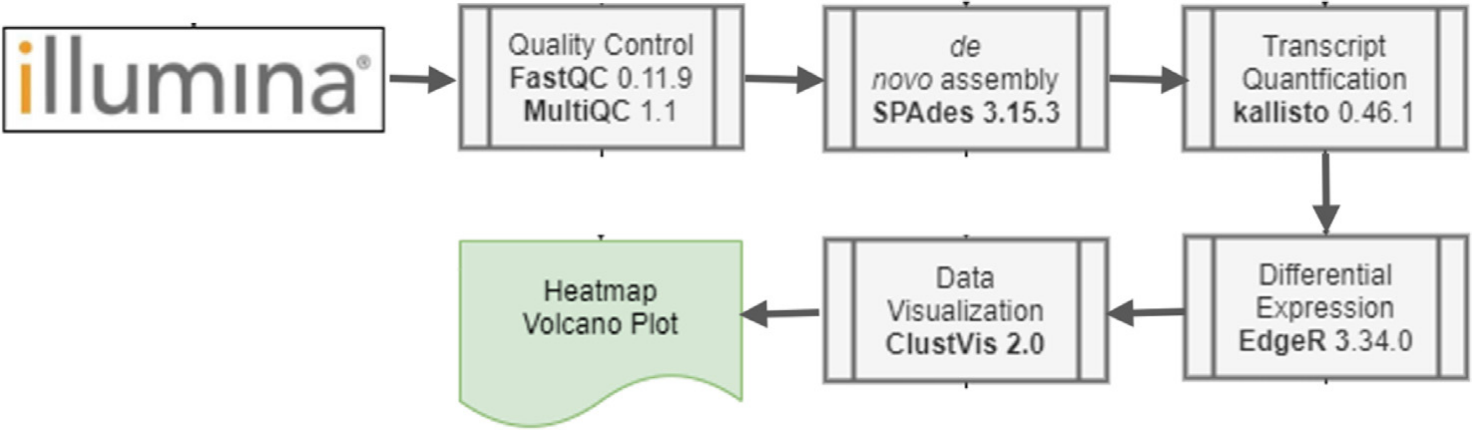
RNA-seq data workflow.

The RNA of EkNS and EkCK weevils (whole body) were extracted using Quick RNA Miniprep Plus Kit (Zymo Research) according to the manufacturer's instructions. The quantity and quality of the total RNA were validated using NanoDrop spectrophotometer (Thermos, USA) for the purity of the RNA samples, and Agilent 2100 Bioanalizer (Agilent RNA 6000 Nano Kit) for the RNA integrity (RIN value), 28S/18S and the fragment length distribution. The samples were further sequenced using Illumina Novaseq 6000 PE150 platform following the steps as follow: a) mRNA enrichment, b) Double stranded cDNA synthesis, c) Repair ends, add A overhang and Adaptor, d) Fragment selection and PCR amplification, e) Library quality test, f) Sequencing on Novaseq 6000 PE150 platform. The workflow for processing RNA-seq data is illustrated in Fig. 1, with the data stored in FASTQ format. Clean reads was obtained by removing the adaptors, unknown bases (N) (more than 10 %) and low quality reads. Those data then ready for further bioinformatics process.

## CRediT authorship contribution statement

**Agus Eko Prasetyo:** Methodology, Software, Writing – original draft, Writing – review & editing. **Edhi Martono:** Conceptualization, Validation, Supervision. **Andi Trisyono:** Visualization, Investigation, Validation. **Alan Soffan:** Conceptualization, Methodology, Software, Writing – review & editing.

## Data Availability

RNAseq of EkCK (Original data) (RNAseq) RNAseq of EkNS (Original data) (RNAseq)

## References

[1] de Chenon R.D. (2016). Proceeding of Sixth IOPRI-MPOB International Seminar of Pests and Diseases, Medan, Indonesia.

[2] Li K., Tscharntke T., Saintes B., Buchori D., Grassa I. (2019). Critical factors limiting pollination success in oil palm: a systematic review. Agric. Ecosyst. Environ..

[3] Rizali A., Rahardjo B.T., Karindah S., Wahyuningtyas F.R., Nurindah B.Sahari, Clough Y. (2019). Communities of oil palm flower-visiting insects: investigating the covariation of *Elaeidobius kamerunicus* and other dominant species. PeerJ.

[4] Lubis F.I., Sudrajat D.Dono (2017). Populasi serangga penyerbuk kelapa sawit *Elaeidobius kamerunicus* Faust dan pengaruhnya terhadap nilai fruit set pada tanah berliat, berpasir dan gambut di Kalimantan Tengah, Indonesia. Jurnal Agrikultura..

[5] Prasetyo A.E., Susanto A. (2012). Serangga penyerbuk kelapa sawit *Elaeidobius kamerunicus* Faust: agresivitas dan dinamika populasi di Kalimantan Tengah. Jurnal Penelitian Kelapa Sawit.

[6] Purba A.R., Prasetyo A.E., Kurniawan A., Supena N., Siregar H.A., Sujadi H.A.Hasibuan, Arif M., Suprianto E. (2016). Proceeding of Sixth IOPRI-MPOB International Seminar of Pests and Diseases, Medan, Indonesia.

[7] Apriyanto A., Tambunan V.B. (2021). Draft genome sequence, annotation, and SSR mining data of *Elaeidobius kamerunicus* Faust., an essential oil palm pollinating weevil. Data Br..

[8] Bakara R.D.J., Tambunan V.B., Apriyanto A., Kusumah Y.M., Sahari B., Buchori D. (2020). Genetic diversity and population structure in *Elaeidobius kamerunicus* (Coleoptera: Curculionidae) inferred from mtDNA COI and microsatellite markers. Adv. Biol. Sci. Res..

[9] Tambunan V.B., Apriyanto A., Ajambang W., Etta C.E., Sahari B., Buchori D., Hidayat P. (2020). Molecular identification and population genetic study of *Elaeidobius kamerunicus* Faust. (Coleoptera: Curculionidae) from Indonesia, Malaysia and Cameroon based on mitochondrial gene. Biodiversitas.

[10] Prasetyo A.E., Purba W.O., Susanto A. (2014). *Elaeidobius kamerunicus*: application of hatch and carry technique for increasing oil palm fruit set. J. Oil Palm Res..

[11] Adaigbe V.C., Odebiyi J.A., Omoloye A.A., Aisagbonhi C.I., Iyare O. (2011). Host location and ovipositional preference of *Elaeidobius kamerunicus* on four host palm species. J. Hortic. Forest..

[12] Hutauruk C.H., Sipayung A., Sudharto (1982). *Elaeidobius kamerunicus* Fst: hasil Uji Kekhususan Inang dan Peranannya Sebagai Penyerbuk Kelapa Sawit. Buletin Pusat Penelitian Marihat.

